# Comparison of Left Ventricular Function Derived from Subject-Specific Inverse Finite Element Modeling Based on 3D ECHO and Magnetic Resonance Images

**DOI:** 10.3390/bioengineering11070735

**Published:** 2024-07-20

**Authors:** Lei Fan, Jenny S. Choy, Chenghan Cai, Shawn D. Teague, Julius Guccione, Lik Chuan Lee, Ghassan S. Kassab

**Affiliations:** 1 Joint Department of Biomedical Engineering, Marquette University and Medical College of Wisconsin, Milwaukee, WI 53233, USA; lei.fan@marquette.edu (L.F.); chenghan.cai@marquette.edu (C.C.); 2 California Medical Innovations Institute, San Diego, CA 92121, USA; jschoy@calmi2.org; 3 Department of Radiology, National Jewish Health, Denver, CO 80206, USA; teagues@njhealth.org; 4 Department of Surgery, University of California at San Francisco, San Francisco, CA 94143, USA; julius.guccione@ucsf.edu; 5 Department of Mechanical Engineering, Michigan State University, East Lansing, MI 48824, USA; lclee@egr.msu.edu

**Keywords:** 3D ECHO, magnetic resonance image, left ventricular mechanics, computational modeling, myocardial contractility

## Abstract

Three-dimensional echocardiography (3D ECHO) and magnetic resonance (MR) imaging are frequently used in patients and animals to evaluate heart functions. Inverse finite element (FE) modeling is increasingly applied to MR images to quantify left ventricular (LV) function and estimate myocardial contractility and other cardiac biomarkers. It remains unclear, however, as to whether myocardial contractility derived from the inverse FE model based on 3D ECHO images is comparable to that derived from MR images. To address this issue, we developed a subject-specific inverse FE model based on 3D ECHO and MR images acquired from seven healthy swine models to investigate if there are differences in myocardial contractility and LV geometrical features derived using these two imaging modalities. We showed that end-systolic and end-diastolic volumes derived from 3D ECHO images are comparable to those derived from MR images ($R^{2}=0.805$ and 0.969, respectively). As a result, ejection fraction from 3D ECHO and MR images are linearly correlated ($R^{2}=0.977$) with the limit of agreement (LOA) ranging from −17.95% to 45.89%. Using an inverse FE modeling to fit pressure and volume waveforms in subject-specific LV geometry reconstructed from 3D ECHO and MR images, we found that myocardial contractility derived from these two imaging modalities are linearly correlated with an $R^{2}$ value of 0.989, a gradient of 0.895, and LOA ranging from −6.11% to 36.66%. This finding supports using 3D ECHO images in image-based inverse FE modeling to estimate myocardial contractility.

## 1. Introduction

Magnetic resonance (MR) imaging is widely accepted as the gold standard for quantifying global and regional heart function and geometrical features, such as left ventricular (LV) mass, volumes, and ejection fraction (EF) in vivo [1]. On the other hand, echocardiographic (ECHO) imaging, which is cost-effective and has a higher temporal resolution than MR imaging, is also widely used to quantify LV function and geometrical features [1]. ECHO imaging can be performed as a 2D acquisition (i.e., transthoracic, TTE, and ECHO) or 3D acquisition (i.e., transesophageal, TEE, and ECHO) that allows full-volume and more accurate acquisition of the LV geometry. Although 3D ECHO imaging enables global and regional LV functions to be assessed more cost-effectively, the discrimination of LV endocardial border using this imaging modality remains challenging, especially for patients with difficult imaging conditions [2]. In connection to this challenge, it is also unclear if LV function and geometry assessed using 3D ECHO are comparable to those assessed using the gold standard MR images.

**Existing studies and challenges**: Previous studies have compared LV function and geometrical features acquired from ECHO with MR images [1,2,3,4]. These features include LV EF, end-systolic volume (ESV) [4], end-diastolic volume (EDV) [3], and myocardial strains [3,4]. Myocardial strains and LV EF are frequently used as a clinical measure of heart function and contractility. These quantities, however, are load-dependent and vary due to many factors, such as a change in preload, afterload, and geometries [5]. Image-based computational models have been developed to estimate load-independent quantities (often in the form of model parameters, i.e., end-systolic elastance, $E_{es}$) that reflect the global and regional LV functions [6,7]. These computer models are typically developed using MR images because of their high spatial resolution in terms of image contrast and boundary detection but computer models based on ECHO images are emerging [8]. Previous studies have not thoroughly compared the myocardial contractility estimated from 3D ECHO images with that derived from MR images using subject-specific inverse FE modeling. It is unclear, however, if the myocardial contractility associated with the LV function estimated from ECHO image-based computer models is comparable to those obtained from MR image-based computer models.

Here, we seek to address this limitation by developing an image-based inverse finite element (FE) modeling framework to assess differences (if any) in the LV contractile function derived from 3D ECHO and MR images acquired from seven normal swine models [9]. We also assess differences in geometrical features such as LV volumes and EF derived from 3D ECHO images with those derived from the respective MR images.

## 2. Methods

### 2.1. Data Acquisition and Preprocessing

All animal experimental data were acquired following the national and local ethical guidelines, including the ARRIVE guidelines, the Guide for the Care and Use of Laboratory Animals, the Public Health Service Policy on Humane Care and Use of Laboratory Animals, the Animal Welfare Act, and an approved Indiana University Purdue University Indianapolis IACUC protocol, regarding the use of animals in research. Statistical power analysis has been performed to determine the required sample size (seven are used in this study). Surgical anesthesia was maintained with isoflurane 1–2%. Ventilation with 100% oxygen was provided with a respirator and PCO_2_ was maintained at approximately 35–40 mmHg. Three-dimensional ECHO images were acquired from seven healthy swine models using a Philips iE33 ultrasound system with the following settings: X3-1 transducer, frame rate of 17–18 Hz, and image depth of 17–19 cm. MR images were obtained from the same swine models using a Siemens 3T Trio MRI scanner (Erlangen, Germany) with the following settings: temporal resolution of 50–100 ms. Long-axis cine steady-state free precession images with a slice thickness of 8 mm were obtained in a radial acquisition with 6 images. Images were obtained both with and without tag lines. The tag lines were at 5 mm increments. Short-axis cine steady-state free precession images with a slice thickness of 5 mm were obtained with a 50% overlap between slices. Images were obtained both with and without grid lines. The grid lines were at 6 mm increments. Three-dimensional ECHO and MR images were acquired with the animals placed in a supine position. The animals were respiratory-gated and both procedures were 3D ECHO and MR images on the same day (3D ECHO first followed by MR images).

In each case, the LV endocardial and epicardial surfaces were manually segmented from the 3D ECHO and MR images associated with the end-diastolic (ED) time point using MeVisLab (MeVis Medical Solutions AG, Bremen, Germany) and TomTec Arena (2014–2020) Imaging Systems GmbH (Philips Healthcare, Andover, MA, USA), respectively. The 3D LV geometry corresponding to the ED time point was reconstructed from these segmented endocardial and epicardial surfaces. The LV endocardial surface was segmented at all time points (160 time points from 3D ECHO images and 15 time points from MR images) in a cardiac cycle from which the LV chamber volume waveform was derived. Together with a pressure waveform measured in normal swine in a previous study [10,11], volume waveforms derived from 3D ECHO and MR images were used to construct the corresponding pressure–volume (PV) loop associated with these two imaging modalities for each swine model.

### 2.2. Left Ventricular Mechanics Finite Element Model

#### 2.2.1. Constitutive Law of the LV

A finite element (FE) mesh was generated in the LV wall defined by the endocardial and epicardial surfaces segmented from the 3D ECHO and MR images for each case. An active stress formulation based on our previous work [12,13,14] was used to describe the mechanical behavior of the LV. In this formulation, the first Piola–Kirchhoff stress tensor ***P*** is decomposed additively into a passive component $P_{p}$ and an active component $P_{a}$ as

(1) $$P=P_{p}+P_{a}$$

The passive stress tensor is defined by $P_{p}=dW/dF$, where ***F*** is the deformation gradient tensor and *W* is a strain energy function of a Fung-type transversely-isotropic hyperelastic material [15] given by

(2) $$W=\frac{1}{2}C\left(e^{Q}-1\right).$$

In Equation (2),

(3) $$Q=b_{ff}E_{ff}^{2}+b_{xx}\left(E_{ss}^{2}+E_{nn}^{2}+E_{sn}^{2}+E_{ns}^{2}\right)+b_{fx}\left(E_{fn}^{2}+E_{nf}^{2}+E_{fs}^{2}+E_{sf}^{2}\right),$$

where *E_ij_* with (*i*, *j*) ∈ (*f*, *s*, *n*) are components of the Green–Lagrange strain tensor E with *f*, *s*, and *n* denoting the myocardial fiber, sheet, and sheet normal directions, respectively. Material parameters of the passive constitutive model are denoted by C, $b_{ff}$, $b_{xx}$, and $b_{fx}$. The active stress is given as $P_{a}=FS_{a}$, where the second Piola–Kirchhoff stress $S_{a}$ is calculated along the local fiber direction using the active stress constitutive relationship [16] as follows:

(4) $$S_{a}=T_{max}(t)\frac{C_{a0}^{2}}{C_{a0}^{2}+EC_{a50}^{2}{(E}_{ff})}e_{f_{o}}⨂e_{f_{0}}$$

In Equation (4), $C_{a0}$ is the peak intracellular calcium concentration, $T_{max}$ is a parameter associated with myocardial contractility that will be estimated, and ${EC}_{a50}$ is the length-dependent calcium sensitivity given by

(5) $${EC}_{a50}=\frac{{{(C}_{a0})}_{max}}{\sqrt{\exp\left(B\left(l-l_{0}\right)\right)-1}}.$$

where B is a material constant, ${{(C}_{a0})}_{max}$ is the prescribed maximum peak intracellular calcium concentration, and ${l=l}_{s0}\sqrt{f_{0}\cdot C{\cdot f}_{0}}$ is the instantaneous sarcomere length based on the prescribed initial length of a sarcomere $l_{s0}$. This constitutive relationship of the LV mechanics described in Equations (1)–(5) will be used in the inverse LV FE model described below.

#### 2.2.2. Finite Element Formulation of the Left Ventricular

The left ventricular base was fixed in the longitudinal direction according to movement out of the plane and the epicardial surface of the LV was constrained using a Robin-type boundary condition with a linear spring. The measured LV pressure and volume were applied as a Neumann condition at the endocardial surfaces. The functional relationship between pressure and volume in the LV is obtained by minimizing a Lagrangian function consisting of a myocardial tissue strain energy function and terms associated with enforcing constraints on myocardial tissue incompressibility, zero-mean rigid body translation and rotation, and cavity volume, such as

(6) $$L\left(u,p,P_{cav},c_{1},c_{2}\right)=\int_{\Omega_{0}}W\left(u\right)dV-\int_{\Omega_{0}}p\left(J-1\right)dV-P_{cav}\left(V_{cav}\left(u\right)-V\right)-c_{1}\cdot \int_{\Omega_{0}}udV-c_{2}\cdot \int_{\Omega_{0}}X\times udV.$$

In Equation (6), u is the displacement field, $P_{cav}$ is the Lagrange multiplier to constraint the cavity volume $V_{cav}(u)$ to a prescribed value V [17], p is a Lagrange multiplier to enforce incompressibility of the tissue (i.e., Jacobian of the deformation gradient tensor J=1), and both $c_{1}$ and $c_{2}$ are Lagrange multipliers to constrain rigid body translation (i.e., zero mean translation) and rotation (i.e., zero mean rotation) [18]. The LV cavity volume $V_{cav}$ is a function of the displacement u and is defined by

(7) $$V_{cav}\left(u\right)=\int_{\Omega_{inner}}dv=-\frac{1}{3}\int_{\Gamma_{inner}}x.nda,$$

where $\Omega_{inner}$ is the volume enclosed by the inner surface $\Gamma_{inner}$ and the basal surface at *z* = 0 and n is the outward unit normal vector.

The pressure–volume relationship of the LV was defined by the solution obtained from minimization of the function. Taking the first variation in the Lagrangian function in Equation (6) leads to the following expression:

(8) $$\delta L\left(u,p,P_{cav},c_{1},c_{2}\right)=\int_{\Omega_{0}}\left(P-pF^{-T}\right):\nabla \delta udV-\int_{\Omega_{0}}\delta p\left(J-1\right)dV-P_{cav}\int_{\Omega_{0}}cof\left(F\right):\nabla \delta udV\;-\delta P_{cav}\left(V_{cav}\left(u\right)-V\right)-{\delta c}_{1}\cdot \int_{\Omega_{0}}udV-\delta c_{2}\cdot \int_{\Omega_{0}}X\times udV-c_{1}\cdot \int_{\Omega_{0}}udV-c_{2}\cdot \int_{\Omega_{0}}X\times \delta udV.$$

which is used in the optimization of myocardial contractility. In Equation (8), P is the first Piola–Kirckhoff stress tensor, F is the deformation gradient tensor, δu,(δp and ${\delta P}_{cav}){\delta c}_{1}\delta c_{2}$ are the variation in the displacement field, Lagrange multipliers for enforcing incompressibility and volume constraint, and zero mean translation and rotation, respectively. The Euler–Lagrange problem then becomes finding $u{\in H}^{1}\left(\Omega_{0}\right),p\in L^{2}\left(\Omega_{0}\right),P_{cav}\in R,c_{1}\in R^{3},c_{2}\in R^{3}$ that satisfies

(9) $$\delta L\left(u,p,P_{cav},c_{1},c_{2}\right)=0$$

and ${\left.u\left(x,y,0\right).n\right|}_{base}=0$ (for constraining the basal deformation to be in-plane) $\forall \delta u{\in H}^{1}\left(\Omega_{0}\right),\delta p\in L^{2}\left(\Omega_{0}\right),{\delta P}_{cav}\in R,{\delta c}_{1}\in R^{3},\delta c_{2}\in R^{3}.$ In the implementation, the displacement field, u, is discretized by quadratic elements and the Lagrange multiplier, p, is discretized by linear elements.

#### 2.2.3. Estimation of the Model Parameters

**Figure 1:**
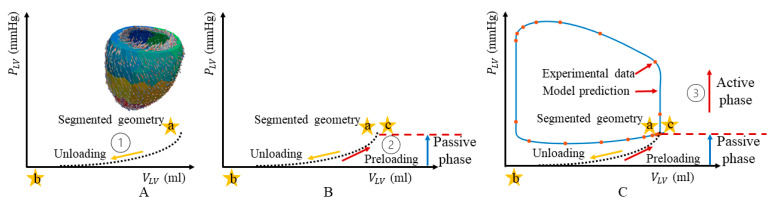
Schematic of the sequential phases in the model parameter estimation process. (**A**) Unloading; (**B**) Passive phase; (**C**) Active phase. a and c denote end-diastolic point. b denotes the LV volume at zero pressure.

The pipeline for estimating parameters by the fitting model predictions to experimental data is divided into two sequential phases associated with the passive and active mechanics (Figure 1).

##### Estimation of Passive Parameters

The LV geometry reconstructed at ED is, in principle, not load-free. Hence, the unloaded (zero pressure) geometry is first determined (points a to b in Figure 1A) using the iterative backward displacement method [19,20]. The passive material parameters C, $b_{ff}$, $b_{xx},$ and $b_{fx}$ in Equation (3) are determined in the unloading process so that the LV FE model-predicted end-diastolic pressure–volume relationship (EDPVR) based on the geometry and measurements derived from 3D ECHO and MR images matches that derived from the single-beat estimation (point b to c in Figure 1B) [21,22].

##### Estimation of the Active Parameter

Once the parameters associated with passive mechanics are estimated, the active stress parameter $T_{max}$ in Equation (4) is estimated by solving a PDE-constrained optimization problem as in our previous work [9,23], where we minimize the cost function representing the mismatch between the simulation and measured data (Figure 1C). The minimization problem is stated as

(10) $$\mathrm{Minimize}\;J(\left(U,p\right),T_{max})\;\;\;\mathrm{subject}\;\mathrm{to}\;\;\;\delta \Pi (U,p)=0.$$

In Equation (10), J is the objective function that is minimized, depending on the state variable displacement U and hydrostatic pressure p, as well as the (control) active stress parameter $T_{max}$ that reflects myocardial contractility. The state variables also depend on the control parameters $\left(U,p\right)=(U(T_{max}),p(T_{max}))$. The constraint $\delta \Pi \left(U,p\right)=0$ in the optimization problem is the Euler–Lagrange equation or the weak formulation of the mechanical equilibrium governing equations [23].

At each time point i, the active stress parameter ${T_{max}}^{i}$ is estimated based on the measured cavity volume $V_{LV}^{i}$ by minimizing the cost function, as follows:

(11) $$J\left(\left(U^{i},p^{i}\right),{T_{max}}^{i}\right)={\left(\frac{P_{LV}^{i}-{\tilde{P}}_{LV}^{i}}{P_{LV}^{i}}\right)}^{2}.$$

The cost function defines the mismatch between simulated cavity pressure ${\tilde{P}}_{LV}^{i}$ and measured cavity pressure $P_{LV}^{i}$ at time point i. Based on this cost function, ${T_{max}}^{i}$ is estimated at each discrete time point i to obtain its corresponding waveform $T_{max}(t)$ over a cardiac cycle.

##### Implementation

The FE model is implemented using the open-source platform FEniCS [24], where the nonlinear systems of equations are solved using Newton’s method and a distributed memory parallel LU solver [25] is used to solve the linear systems. The cost function in Equation (11) is minimized using a gradient-based bound-constrained Broyden–Fletcher–Goldfarb–Shanno (L-BFGS) optimization algorithm [26], where the gradient is computed from an adjoint-state method [9,23] using dolfin-adjoint [27]. The initial guess of the contractility is set to be 0 in the first iteration. The simulation is run until the relative error between the model predictions and experimental measurements in Equation (11) decreases below 5%. Details of the model parameter estimation approach can be found in [9,23].

### 2.3. Statistical Analysis

The comparison between quantities derived from 3D ECHO and MR images was assessed by performing a linear regression analysis from which the gradient and coefficient of determination $R^{2}$ were computed. The coefficient of determination $R^{2}$ in a regression analysis explains the variability of each quantity (i.e., LV EDV, LV ESV, EF, and myocardial contractility) derived from 3D ECHO and MR images. Bland and Altman’s analysis [28] was performed to assess the difference between quantities derived from 3D ECHO and MR images, i.e., bias and root mean square (rms) differences and limit of agreement (LOA). All data are expressed as mean ± SD.

### 2.4. Scientific Contribution

This study integrates the inverse FE modeling with 3D ECHO and MR images to compare the accuracy of LV geometry, volumes, and model-predicted myocardial contractility. Accurate assessment of LV myocardial contractility is crucial for disease diagnosis and treatment development. By validating the FE models derived from 3D ECHO and MR images, this study seeks to build a robust framework for estimating myocardial contractility with different imaging modalities.

## 3. Results

### 3.1. Left Ventricular Wall Thickness and Volumes

**Figure 2:**
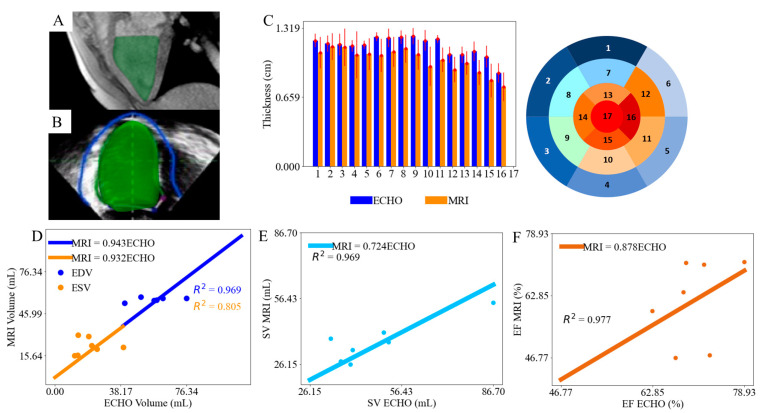
Segmented LV endocardial surfaces in (**A**) MR images and (**B**) 3D ECHO images. (**C**) Comparison of regional LV wall thickness derived from 3D ECHO and MR images; Correlations of (**D**) LV EDV and ESV; (**E**) SV and (**F**) EF derived from 3D ECHO and MR images.

Segmented LV endocardial surfaces corresponding to the 3D ECHO and MR images are shown in Figure 2A,B, respectively. The average wall thickness in each segment is calculated. Both 3D ECHO and MR images show that the myocardial wall thickness is more homogeneous (1.17 ± 0.049 cm in 3D ECHO and 1.099 ± 0.123 cm in MR images) at the mid-wall and basal regions (region 1 to 12) compared to the apical region (region 13 to 17), where it is less homogenous and thinner (varying between 0.89 and 1.06 cm in 3D ECHO and 0.76–0.98 cm in MR images) (Figure 2C). Septal regions (regions 2, 3, 8, 9, and 14) have a thicker wall than the free wall regions (regions 5, 6, 11, 12, and 16) (Figure 2C). Left ventricular geometries segmented from 3D ECHO images have a thicker wall than those segmented from the MR images (Figure 2C). An outlier of LV EDV in case 1 that lies an abnormal distance from other values of the dataset as determined by the median and quartile range was identified, where the LV EDV difference between 3D ECHO and MR images was 49.9 mL, significantly deviating from the median range of [−16.5, 27.6] [29]. This outlier of LV volume might be caused by the manual segmentation of geometry. This data point was therefore excluded from the linear regression analysis as the inclusion of outliers impacts the accuracy of statistical analysis. The analysis revealed that MR and 3D ECHO image-derived LV volumes are positively correlated (EDV: gradient 0.943, $R^{2}$ value 0.969; ESV: gradient 0.932, $R^{2}$ value 0.805) (Figure 2D). The resultant stroke volume (SV) and ejection fraction (EF) derived from MR and 3D ECHO images are also positively correlated with gradients 0.724 ($R^{2}$ value 0.969) and 0.878 ($R^{2}$ value 0.977), respectively (Figure 2E,F).

**Figure 3:**
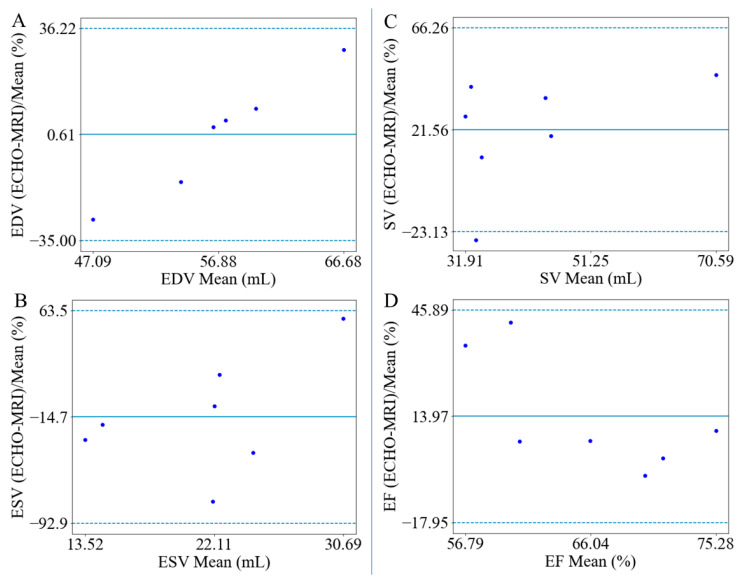
Bland–Altman analyses of the percentage difference in (**A**) EDV, (**B**) ESV, (**C**) SV, and (**D**) EF based on 3D ECHO and MR images.

Bland–Altman analyses show that the relative differences of the LV EDV, ESV, SV, and EF estimated between 3D ECHO and MR images all fell within the 95% confidence interval (Figure 3). An outlier of LV EDV in case 1 was removed for analysis as determined by the median and quartile range [29,30]. It shows that the mean LV EDV estimated based on the 3D ECHO images is greater than that based on the MR images by 0.61%, with an LOA ranging from −35.00% to 36.22% (Figure 3A), whereas the mean LV ESV estimated based on the 3D ECHO images is smaller than that based on the MR images by 14.7% with an LOA ranging from −92.92% to 63.48% (Figure 3B). Bland–Altman analyses also show that SV and EF derived from the 3D ECHO images are both larger (by 21.56% and 13.97%) compared to those derived from MR images with the LOA ranging from −23.13% to 66.26% and from −17.95% to 45.89%, respectively. The relative difference between these 2 quantities (SV and EF) derived from 3D ECHO and MR images all fell within the 95% confidence interval (Figure 3C,D).

### 3.2. Left Ventricular Pressure–Volume Loops

**Figure 4:**
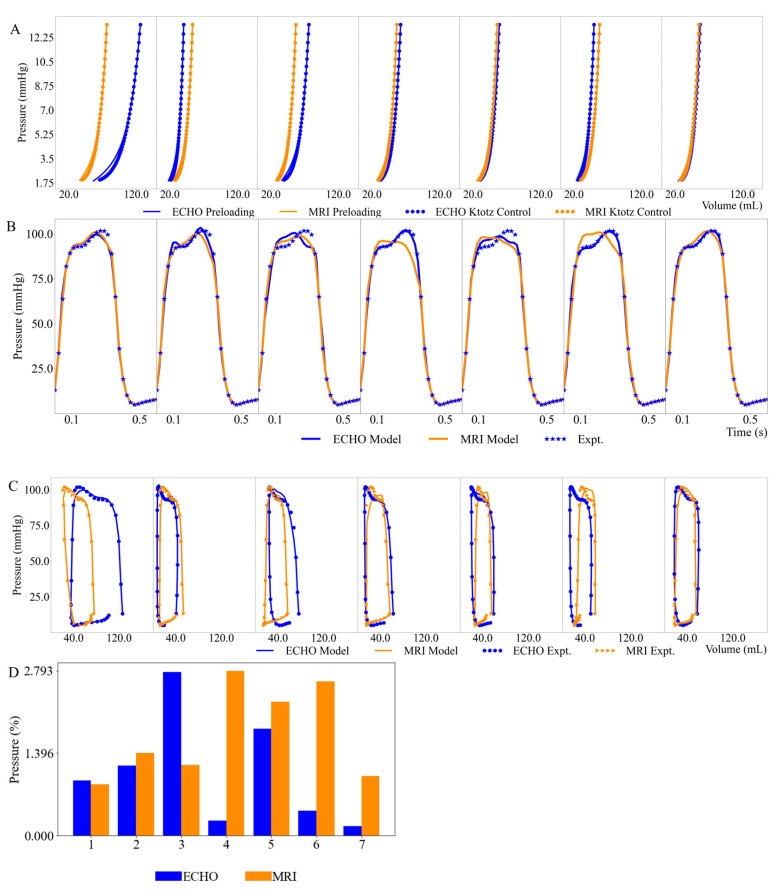
Comparison of (**A**) model predicted EDPVRs and Klotz curves; (**B**) Model predicted and measured LV pressure waveforms; (**C**) Model predicted and measured LV PV loops. (**D**) Relative errors between model predictions and measurements of LV pressures for all cases. Blue and orange solid lines denote the model predicted PV loops based on 3D ECHO and MRI, respectively. Blue and orange dots denote the experimentally measured PV loops based on 3D ECHO and MRI, respectively.

The model-predicted EDPVRs based on geometries and EDVs derived from 3D ECHO and MR images of the animals are consistent with those obtained from the single-beat estimation based on the Klotz relationships (Figure 4A). Fitted LV pressure waveforms and PV loops based on geometries and volume waveforms derived from 3D ECHO and MR images are also in good agreement with the experimental measurements (Figure 4B,C). The relative error between model predictions and experimental measurements of LV pressure based on Equation (11) is below 5% in all seven cases (Figure 4D).

### 3.3. Myocardial Contractility

**Figure 5:**
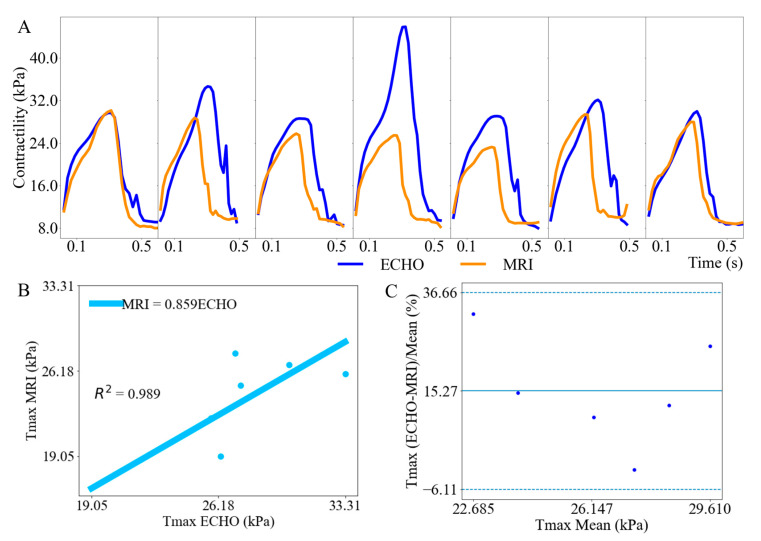

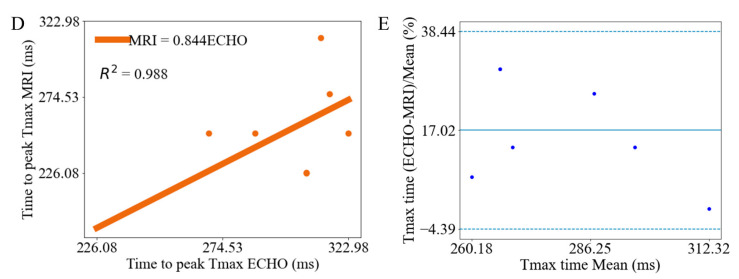
(**A**) Comparison of $T_{max}$ waveforms derived from 3D ECHO and MR images. (**B**) Correlation of peak $T_{max}$ derived from 3D ECHO and MR images. (**C**) Bland–Altman analysis of peak $T_{max}$. (**D**) Correlation of model-predicted time to reach peak $T_{max}$ derived from 3D ECHO and MR images. (**E**) Bland–Altman analysis of time to peak $T_{max}$.

**Figure A1:**
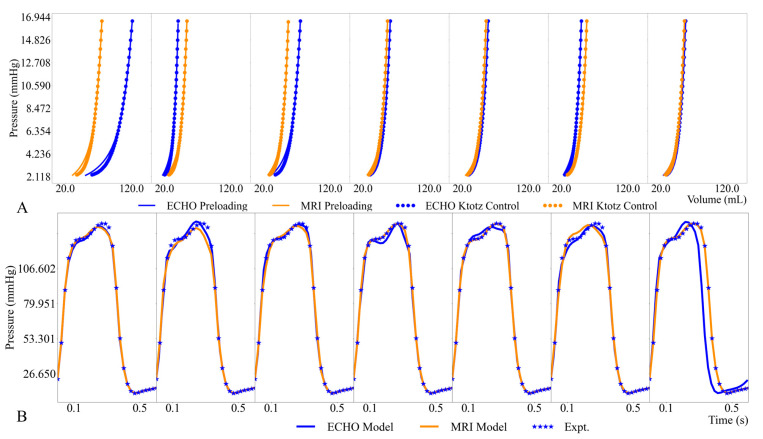

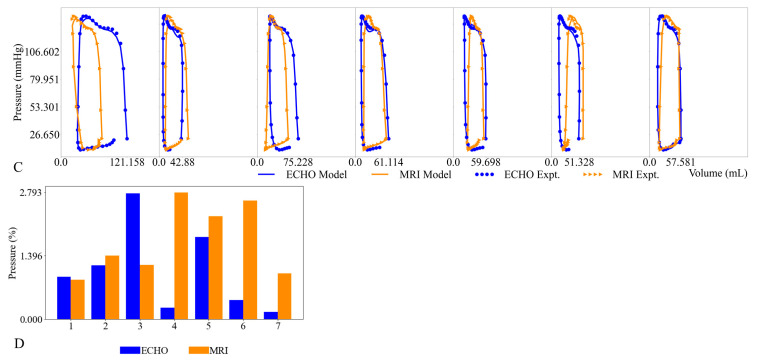
Comparison of (**A**) model-predicted EDPVRs and Klotz curves; (**B**) Model-predicted and measured LV pressure waveforms; (**C**) Model-predicted and measured LV PV loops. (**D**) Relative errors between model predictions and measurements of LV pressures for all cases. Blue and orange solid lines denote the model-predicted PV loops based on 3D ECHO and MRI, respectively. Blue and orange dots denote the experimentally measured PV loops based on 3D ECHO and MRI, respectively.

**Figure A2:**
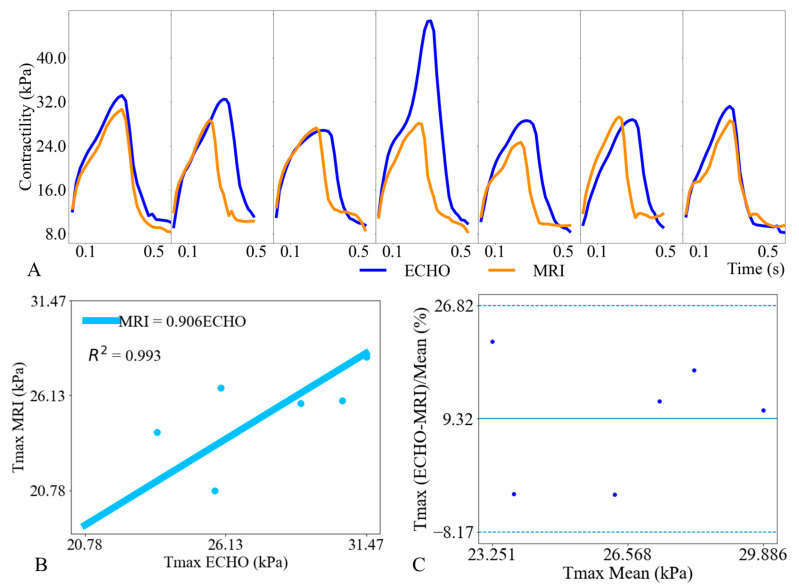
(**A**) Comparison of $T_{max}$ waveforms derived from 3D ECHO and MR images. (**B**) Correlation of peak $T_{max}$ derived from 3D ECHO and MR images. (**C**) Bland–Altman analysis of peak $T_{max}$.

The model-predicted waveforms of the active stress parameter $T_{max}$ associated with myocardial contractility derived from 3D ECHO images are comparable to those derived from MR images except for case 4 (Figure 5A). The outlier case 4 was removed from the linear regression analysis as determined by the median and quartile range [29,30]. The reason for the outlier might be from manual segmentation. The linear regression analysis revealed that the peak $T_{max}$ derived from MR images is positively correlated to those derived from 3D ECHO images with a gradient of 0.859 and $R^{2}$ value of 0.989 (Figure 5B). Bland–Altman analysis shows that the relative difference in peak $T_{max}$ estimated from 3D ECHO and MR images is within the 95% confidence interval with an LOA ranging from −6.11% to 36.66%. (Figure 5C). Time to peak $T_{max}$ derived from MR images is positively correlated to those derived from 3D ECHO images with a gradient of 0.844 and an $R^{2}$ value of 0.988 (Figure 5D). The corresponding Bland–Altman analysis also shows that the relative difference in time to peak $T_{max}$ estimated from 3D ECHO and MR images is within the 95% confidence interval with an LOA ranging from −4.39% to 38.44% (Figure 5E). We note that the LV pressure waveform used in seven cases (Figure 4B) was measured from normal swine in our previous study [10], as we stated in Section 2.1. To test the effects of different LV pressure waveforms on the model-predicted contractility, sensitivity analyses using a different LV pressure waveform were performed (Appendix A). The model-predicted contractility correlation using the two different LV pressure waveforms is comparable (with a linear gradient of 0.859 and 0.906, respectively) (Figure A1 and Figure A2). Although the LV pressure waveform is identical for swine and although the LV volume could be derived based on 3D ECHO or MR images, the sensitivity analysis shows that it does not affect the comparison of model-predicted contractility based on measurements from 3D ECHO and MR images.

## 4. Discussion

The *key* finding is that the myocardial contractility $T_{max}$ derived from the inverse FE computer modeling using 3D ECHO and MR images are comparable with a mean difference of 3.94 ± 3.02 kPa and LOA ranging from 1.45 to 9.34 kPa. Other specific findings include the following. *First*, the LV wall is thicker in the geometry reconstructed from 3D ECHO images than from MR images by 9.10%. *Second*, the LV EDV derived from 3D ECHO images is greater than those derived from MR images by 2.49% whereas the LV ESV derived from 3D ECHO is smaller than that derived from MR images by 9.25%. Overall, the findings demonstrate that using 3D ECHO images in inverse FE modeling approaches to estimate myocardial contractility is robust and yields comparable results to those using MR images.

### 4.1. Left Ventricular Geometry and Volumes

The analyses in this study are comparable to previous analyses (Table 1). Specifically, our analyses show that EDV, ESV, SV, and EF segmented from 3D ECHO and MR images are positively correlated with linear gradients of 0.943, 0.932, 0.724, and 0.878, respectively (Figure 2D–F). These values compare well with those found in other studies, which also found that LV volumes estimated from 3D ECHO images are smaller compared to those estimated from MR images with gradients (from linear regression analysis) ranging between 0.86 to 0.88 for LVEDV, 0.88 to 0.96 for LVESV [24,31], and 0.87 for LV EF [32]. Differences in EDV, ESV, and EF derived from the two imaging modalities are 2.5%, 9.1%, and 10%, respectively. Our findings of 1.40 mL and 2.08 mL differences in the LV EDV and LV ESV between 3D ECHO and MR imaging (Figure 2D) are comparable to the differences of 4.00 mL reported in previous clinical studies [33]. Our finding of an 8.48% difference in LV EF between 3D ECHO and MR imaging (Figure 2F) is within the range found in previous studies, which reported differences of 5.42–15.00% [33,34]. We note that other studies have also found that the LV volumes estimated from 3D ECHO images are smaller than those estimated from MR images, with differences ranging from −4 ± 43 mL [32] to −41 ± 37 mL for LV EDV [35,36] and from 0 ± 33 mL [32] to −34 ± 45 mL [37] for ESV. Although our analyses show that the LV EDV estimated from 3D ECHO is greater compared to those estimated from MR images by 1.40 mL, this difference still falls within these ranges [29,31]. Our analyses also show that LV ESV from 3D ECHO is smaller than from MR images by 2.08 mL, which agrees with previous studies [29,32].

The coefficient $R^{2}$ values associated with the correlation of volume between those estimated from 3D ECHO and MR images found in previous studies (LV EDV: 0.929 and LV ESV: 0.971) [38] are also comparable to those found here (LV EDV: 0.969 and LV ESV: 0.805). Other studies found that the $R^{2}$ value varies from 0.94 [2] to 0.99 [39,40] for LV EDV, from 0.93 [2] to 0.99 [39] for LV ESV, and from 0.93 [2] to 0.98 [39] for LV EF. Our analyses in terms of the $R^{2}$ value for LV EDV (0.969), LV ESV (0.805), and LV EF (0.977) are comparable with these ranges.

The LOAs associated with the volume differences between those estimated from 3D ECHO and MR images are comparable to previous findings. Since most previous studies analyzed the LOAs in terms of volume difference instead of relative differences of volumes (Figure 3 and Figure 5), LOAs of volume difference are reported for comparison here. Our analyses show that the LOAs range from −19 to 22 mL for the LV EDV, from −22 to 18 mL for the LV ESV, and from −10 to 27% for the LV EF, respectively, which is comparable to previous analyses ranging from −57 to 47 mL for the LV EDV, from −58 to 46 mL for the LV ESV, and from −8.3 to 7.7% for the LV EF, respectively [2].

### 4.2. Left Ventricular Function

Myocardial strain derived from 3D ECHO and MR images is increasingly used as an index to assess LV function and myocardial contractility [3,4]. Myocardial strain, however, is a load-dependent metric affected by preload and afterload [41]. Image-based inverse FE computer modeling approaches have been developed to circumvent this issue to estimate load-independent metrics associated with myocardial contractility [8,36,37,38,39]. Image-based FE models are developed mostly based on MR images [42,43,44,45]. For example, an image-based joint state-parameter estimation method was developed to estimate tissue contractility by matching the model-predicted surface contours at end-systole to that segmented from the MR images from pigs [46]. To the best of our knowledge, inverse FE modeling to estimate myocardial contractility has only been performed based on regional strains using 3D ECHO images but the unloaded geometry is reconstructed from MR images [47]. Furthermore, although LV geometries together with LV volumes have been compared by reconstructing the geometries based on 3D ECHO and MR images [48,49,50], it has not been established as to whether myocardial contractility derived from inverse FE modeling based on 3D ECHO images is comparable to that derived from MR images. This knowledge gap is filled here, showing that myocardial contractility (as indexed by the model parameter $T_{max}$) estimated in an inverse FE modeling based on 3D ECHO and MR images are comparable and highly correlated (Figure 5). This is also the key novelty of this study. Specifically, model predictions show that the peak myocardial contractility $T_{max}$ derived from MR images is linearly correlated with those derived from 3D ECHO images with a gradient of 0.859 and $R^{2}$ value of 0.989. The mean difference in peak $T_{max}$ derived from 3D ECHO and MR images is 3.94 kPa and the relative difference is 17%. This finding supports using 3D ECHO in image-based inverse FE modeling to estimate myocardial contractility. This finding is also significant because 3D ECHO imaging is more cost-effective and accessible, especially because patients implanted with medical devices (e.g., left ventricular assist devices and pacemakers) are typically contraindicated for MR imaging.

## 5. Limitations

There are some limitations in this study. First, myocardial strains in the LV are not considered in the inverse FE modeling framework, as only cine MR images are acquired. Future studies may include regional strains estimated directly using 3D ECHO [47] and tagged MR images or indirectly using cine MR images with feature-tracking methods such as the hyperelastic wrapping method [51]. Second, subject-specific pressure waveform is not available. The pressure waveform used in all cases was obtained from a healthy swine in a separate study. Nevertheless, we have performed a sensitivity analysis and shown that the myocardial contractility derived from 3D ECHO and MR images with a different pressure waveform is comparable. Third, 3D ECHO and MR images were acquired only from healthy swine models in this study, without considering animal models with diseases. This limits the generalizability of the findings to diseased conditions. Future research will apply this methodology to diseased animal models to compare the model-predicted contractility based on different imaging modalities under various pathological conditions. Such studies could provide more comprehensive insights into the applicability and robustness of the inverse FE modeling framework in clinical settings.

## 6. Conclusions

In summary, myocardial contractility $T_{max}$ estimated using inverse FE modeling and LV volumes computed from segmented geometries reconstructed from 3D ECHO and MR images are comparable with a mean difference of 3.94 ± 3.02 kPa. Specifically, the LV wall thickness and LV EDV and LV ESV derived from 3D ECHO and MR images are comparable with a percentage difference of less than 10%. These findings support the application of 3D ECHO images in subject-specific inverse FE modeling frameworks to estimate cardiac parameters. In the future, myocardial strain measurements using tagged MR images or feature-tracking methods will be integrated into the inverse FE modeling framework. Subject-specific pressure waveforms will be collected and utilized to improve the accuracy of contractility estimations. This study will be extended by including animal models with various cardiac pathologies to validate the robustness of the algorithm. Clinical trials will also be potentially conducted by integrating patient-specific imaging and hemodynamic measurements to validate the performance of the algorithm in human subjects with different cardiac conditions.

## Figures and Tables

**Table 1:** Comparison between this analysis and those from previous studies.

	This Study				Previous Studies			
	Linear Gradient	MR Images-3D ECHO	$R^{2}$ Value	LOAs	Linear Gradient	MR Images-3D ECHO	$R^{2}$ Value	LOAs
LVEDV	0.943	1.40 mL	0.969	−19~22 mL	0.86–0.88 [24,31]	4.00 mL [33]	0.929–0.99 [2,38,39,40]	−57~47 mL [2]
LVESV	0.932	2.08 mL	0.805	−22~18 mL	0.88–0.96 [24,31]	4.00 mL [33]	0.93–0.99 [2,38,39]	−58~46 mL [2]
EF	0.878	8.48%	0.977	−10~27%	0.87 [32]	5.42–15.00% [33,34]	0.93–0.98 [2,39]	−8.3~7.7% [2]

## Data Availability

The raw data supporting the conclusions of this article will be made available by the authors on request.

## Appendix A

A sensitivity analysis was performed to test the effects of LV pressure waveform on myocardial contractility derived from 3D ECHO and MR images. The model-predicted EDPVR derived from 3D ECHO and MR images match well with the Klotz curve (Figure A1A). Model-predicted LV pressure waveforms and PV loops agree with the measurements (Figure A1B,C), with a relative error below 5% (Figure A1D). Figure A2A shows a comparison of model-predicted contractility derived from 3D ECHO and MR images. The linear regression analysis revealed that the peak $T_{max}$ derived from MR images is positively correlated with those derived from 3D ECHO images with a gradient of 0.906 and $R^{2}$ value of 0.993 (Figure A2B). Bland–Altman analysis shows that the relative difference in peak $T_{max}$ estimated from 3D ECHO and MR images is within the 95% confidence interval with an LOA ranging from −8.17% to 26.82%. (Figure A2C). Model-predicted contractility based on different pressure waveforms (Figure 5 and Figure A2) shows the consistent correlation between those derived from 3D ECHO and MR imaging.
